# Ensuring good quality rna for quantitative real-time pcr isolated from renal proximal tubular cells using laser capture microdissection

**DOI:** 10.1186/1756-0500-7-62

**Published:** 2014-01-27

**Authors:** Jie Yin Yee, Lie Michael George Limenta, Keith Rogers, Susan Mary Rogers, Vanessa SY Tay, Edmund JD Lee

**Affiliations:** 1 Department of Pharmacology, Yong Loo Lin School of Medicine, National University of Singapore, MD11 10 Medical Drive #05-09, Singapore 117597, Singapore; 2 IMCB Histopathology Laboratory, 61 Biopolis Drive, Proteos Building Level 6, Singapore 138673, Singapore

## Abstract

**Background:**

In order to provide gene expression profiles of different cell types, the primary step is to isolate the specific cells of interest via laser capture microdissection (LCM), followed by extraction of good quality total RNA sufficient for quantitative real-time polymerase chain reaction (qPCR) analysis. This LCM-qPCR strategy has allowed numerous gene expression studies on specific cell populations, providing valuable insights into specific cellular changes in diseases. However, such strategy imposed challenges as cells of interests are often available in limited quantities and quality of RNA may be compromised during long periods of time spent on collection of cells and extraction of total RNA; therefore, it is crucial that protocols for sample preparation should be optimised according to different cell populations.

**Findings:**

We made several modifications to existing protocols to improve the total RNA yield and integrity for downstream qPCR analyses. A modified condensed hematoxylin and eosin (H&E) staining protocol was developed for the identification of rat renal proximal tubular cells (PTCs). It was then determined that a minimal of eight thousands renal PTCs were required to meet the minimal total RNA yield required for downstream qPCR. RNA integrity was assessed using at every progressive step of sample preparation. Therefore, we decided that the shortened H&E staining, together with microdissection should be performed consecutively within twenty minutes for good quality for gene expression analysis. These modified protocols were later applied on six individual rat samples. A panel of twenty rat renal drug transporters and five housekeeping genes showed Ct values below thirty-five, confirming the expression levels of these drug transporters can be detected.

**Conclusions:**

We had successfully optimized the protocols to achieve sufficient good quality total RNA from microdissected rat renal PTCs for gene expression profiling via qPCR. This protocol may be suitable for researchers who are interested in employing similar applications for gene expression studies.

## Findings

### Background

Gene expression studies of isolated tissues are often confounded by the reality that such tissues are made up of highly heterogeneous cell types. The relative difficulty in obtaining a pure population of cells has often imposed a huge challenge for researchers as manual isolation of cells is often laborious, requires long working hours and complicated procedures [1]. Furthermore, after such laborious extractions, there is often doubt about the quality of RNA intended for gene expression studies. The introduction of LCM provided a breakthrough that promised easy identification and collection of specific cell types [2-4]. However, there were several concerns with regards to achieving sufficient good quality RNA for downstream qPCR.

LCM often requires several upstream procedures such as tissue collection, cryosectioning and staining prior to microdissection. The majority of these preparations are performed at room temperature, leading to possible deleterious effects on the RNA quality by the presence of RNases. Furthermore, microdissected cells often come in small numbers which may not be sufficient for commercialised qPCR kits. Such difficulties may lead to biased expression profiling and missing information, especially for rare transcripts.

While detailed reports had been described for specific cell population expression profiling studies, data about RNA quality assurance specifically for cells harvested through LCM are seldom available in the literature [5-12]. It had been reported that different tissues may have varying levels of RNases present and different cell types will yield different amounts of RNA; therefore, protocols should be developed and/or fine tune for different cell populations [13].

In this study, we looked into the collection of rat renal proximal tubular cells (PTCs) using LCM, and also evaluated several aspects during sample preparation to ensure total RNA isolated is of good quality and quantity for downstream qPCR analysis. The optimized protocols were then applied onto a group of six rats for the gene expression profiling of twenty drug transporters and five housekeeping genes.

### Methods

#### RNase-free experimental environment

All procedures were performed in an RNase-free environment. RNase decontamination wipes (RNaseZap, Ambion) were used as a cleaning agent for removing RNase for pipettors and countertops, nuclease-free 70% ethanol, and pipette tips with filters were used.

#### Tissue preparation

Rats were euthanized via carbon dioxide asphyxiation. Both kidneys were removed immediately after death and middle cross-section was sectioned using a sterile blade. Sectioned kidneys were wrapped in aluminium foil and frozen in liquid nitrogen immediately. Frozen kidneys were then transferred to Leica Cryostat CM3050S (Leica Biosystems) at −16°C. Kidneys were then embedded onto block holder using Tissue-Tek® OCT™ Compound (Sakura, The Netherlands). The embedded kidneys were sectioned thrice with 10 μm in thickness. The kidney sections were then gently transferred onto PEN membrane glass slides. The slides containing kidney sections were allowed to dry in cryostat for 2 minutes before histology (H&E) staining.

#### Hematoxylin and eosin staining

All procedures were performed on wet ice to minimize RNase activity. Table 1 lists the difference in the steps between the standard and modified modified H&E staining protocol. All reagents were freshly prepared and filtered on the day of usage and only nuclease-free water was used when necessary. Step 1 of staining (drying of slides) was performed in cryostat at.

**Table 1:** A comparison between standard and modified H&E staining protocol durations

**Standard H&E staining**			**Modified H&E staining**		
**Steps**	**Reagent**	**Time**	**Steps**	**Reagent**	**Time**
1	Haematoxylin 2	2 mins	1	Air dry slides in cryostat at −16°C	2 mins
2	Gentle running water	2 mins	2	70% Ethanol	30 secs
3	Clarifier 2	2 mins	3	Water	5 secs
4	Gentle running water	2 mins	4	Hematoxylin 2	10 secs
5	Bluing solution	2 mins	5	Bluing reagent	10 secs
6	Gentle running water	2 mins	6	70% Ethanol	10 secs
7	70% Ethanol	2 mins	7	Eosin/Phloxine	2 secs
8	Eosin/Phloxine	15 secs	8	100% Ethanol	5 secs
9	70% Ethanol	2 mins	9	100% Ethanol	30 secs
10	95% Ethanol	2 mins	10	100% Ethanol	30 secs
11	100% Ethanol	2 mins	11	Xylene	5 secs
12	100% Ethanol	2 mins	12	Xylene	30 secs
13	100% Ethanol	2 mins	13	Xylene	1 min
14	Xylene	2 mins	Total time		~5 mins
Total time		~26 mins			

#### Collection of rat renal PTCs via LCM

H&E stained slides were placed on Arcturus^XT^™ LCM instrument (Applied Biosystems, USA) modular stage. Illumination contrast and desired magnification objective were adjusted for optimal visualisation. Using simple drawing tools supplied, renal PTCs or desired cells were outlined on monitor screen for microdissection. After dissection, renal PTCs or desired cells that were captured and transferred onto CapSure® HS LCM Caps (Invitrogen, USA) were inspected under microscope for reconfirmation.

#### Modified total RNA isolation and DNase I treatment

Total RNA was isolated immediately after microdissected renal PTCs. PicoPure® RNA Isolation Kit (Applied Biosystems®) was used for the total RNA extraction according to manufacturer’s protocol with several modifications.

Briefly, microdissected PTCs on caps and ExtracSure Extraction Device was assembled. This assembly was placed in a HS Alignment Tray and 30 μL of extraction buffer was gently pipetted into the well and the alignment tray was covered with a pre-warmed incubation block. The assembly was then incubated for 30 minutes at 42°C. Microcentrifuge tube with CapSure-ExtracSure assembly was centrifuged at 1000 g for 2 minutes for collection of cell lysate to the bottom of centrifuge tube. The RNA purification column was pre-conditioned by adding 250 μL of conditioning buffer before incubating at ambient temperature 5 minutes. The RNA purification column was then centrifuged at 16, 000 g for 1 minute for removal of conditioning buffer. 30 μL of 70% ethanol was then added to the cell extract and mixture was mixed well by pipetting up and down gently. The cell extract-70% ethanol mixture was transferred to RNA purification column and centrifuged at 100 g for 2 minutes for RNA binding, followed by a quick spin at 16, 000 g for 30 seconds to remove flowthrough. This step was repeated twice to ensure maximal RNA binding to the column membrane. 100 μL washing buffer 1 was pipetted to the same RNA Purification Column and centrifuged for 1 minute at 8, 000 g. The bound RNA was later treated with DNAse I (RNase-Free DNase Set, Qiagen). 10 μL of DNAse I stock solution was added to 30 μL RDD Buffer and mixed well via gently pipetting. This diluted DNAse I-RDD mixture was then added to RNA Purification Column containing bound total RNA and allowed to stand in ambient temperature for 30 minutes. 40 μL Wash Buffer 1 was added to the column and centrifuged at 8, 000 g for 15 seconds to stop the treatment. The DNAse I treated RNA was washed twice with 100 μL of Wash Buffer 2. The purification column was transferred to a new 0.5 mL microcentrifuge tube provided and 11 μL elution buffer was added. The tube assembly was left to incubate at room temperature for 1 minute and centrifuged at 1, 000 g for 1 minute to distribute elution buffer in the column, followed by spinning at 16, 000 g for 1 minute to RNA elution. The eluted RNA was then transferred back to the same purification column and elution was repeated twice to maximize total RNA yield.

#### RNA quality control

##### RNA integrity number (RIN)

RIN was measured using Agilent 2100 Bioanalyzer with Agilent RNA 6000 Pico Kit. All procedures were performed according to manufacturer’s protocol.

##### Total RNA concentration and purity ratios

Total RNA concentrations and purity ratios (260/280 and 260/230) were measured using NanoDrop 2000 UV–vis Spectrophotometer (Thermo Scientific) unless otherwise stated.

#### Gene expression analysis

Total RNA was then converted to complementary DNA (cDNA) using RT2 PreAMP cDNA Synthesis Kit (SABiosciences, Qiagen) according to manufacturer’s protocol. Freshly synthesized cDNA then underwent one round of amplification (RT2 PreAMP PCR Mastermix and RT2 PreAMP Pathway Primer Mix – CAPR09572) before qPCR. Using customized PCR array plates from SABiosciences (Plate ID – CAPR09572), preamplified cDNA was added into each well containing specific primers for genes of interest. DNA Engine Opticon-2 (BioRad) was used for qPCR.

### Results

In order to optimise the protocol for the collection of good quality RNA for qPCR, we expanded the following: (a) suitability of modified H&E staining on the identification of rat renal proximal tubular cells (PTCs); (b) the minimal number of rat renal PTCs required to yield sufficient total RNA for downstream qPCR; (c) the quality of total RNA, (d) modifications made to DNase I treatment and lastly, (e) the application of isolated total RNA on qPCR analyses.

#### Modified H&E staining

**Figure 1:**
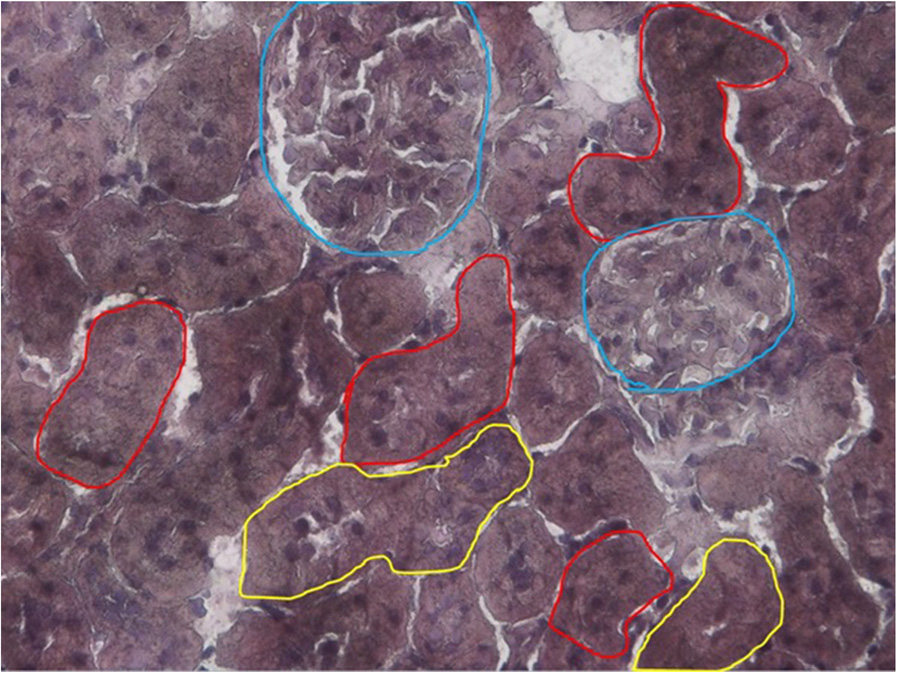
**Identification of rat renal PTCs via modified H&E staining protocol.** The shortened H&E staining procedure did not compromise on the identification of rat renal PTCs. The rat renal PTCs were recognized by their distinct brush border membranes and were circled in red. Renal distal tubular cells and glomerulus were outlined in yellow and blue respectively.

In order to maximise the time for collection of renal cells via LCM, we used a shortened H&E staining protocol to minimise the degradation of RNA by surrounding RNases. Although the original H&E protocol of approximately twenty-six minutes was condensed to a five-minute protocol, the modified H&E staining protocol was adequate for the identification of rat renal PTCs against other renal cells. The rat renal PTCs were identified via their unique brush border membranes and were selected in red in Figure 1.

#### Minimum number of rat renal PTCs required to yield sufficient total RNA for downstream qPCR

There have been a number of reports on the various number of cells required for downstream gene expression analyses. These ranged from single cell studies on traumatic brain injury, ten avian embryo to less than five thousand breast cancer cells [14-16]. However, such information may not be applicable on all tissue types. Therefore, the minimal number of renal tubular cells required for downstream qPCR will have to be determined. We collected three sets of renal cells three thousand, eight thousand and eighteen thousand renal cells respectively. Total RNA was then isolated using PicoPure® RNA Isolation Kit (Life Technologies) according to the manufacturer’s protocol with some modifications. The RNA concentrations were measured using Agilent Bioanalyzer RNA 6000 Pico kit.

**Figure 2:**
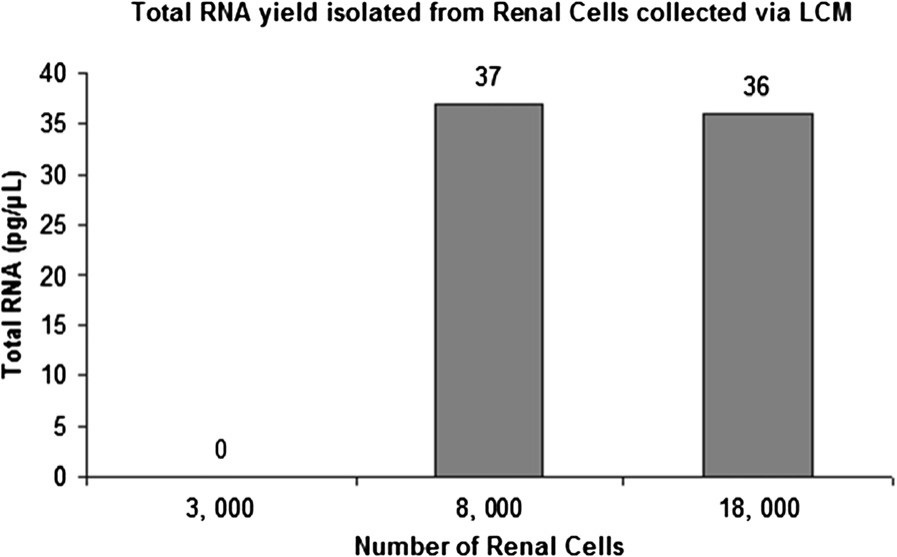
**Total RNA concentrations from different number of renal cells.** Total RNA concentrations were measured using Agilent 2100 Bioanalyzer with Agilent RNA 6000 Pico Kit. It was observed that 3000 renal cells yielded a total RNA concentration too low to be detected while 8000 and 18000 renal cells produced similar total RNA yields.

As seen in Figure 2, we observed the total RNA extracted from three thousand renal cells was too low to be detected. The total RNA concentrations yielded from eight thousand and eighteen thousand renal cells were 37 pg/μL and 36 pg/μL respectively which were adequate for qPCR application. These similar yields may suggest there was no apparent advantage in attempting collection of more than eight thousand renal cells. The reason for this is not clear but may be related to the capacity limit of the total RNA isolation kit.

#### Quality of total RNA

It was previously reported that stained cryosections should undergo LCM immediately to prevent RNA degradation [17]. Although this approach is ideal, it is sometimes impracticable because of time constraints. We observed minimal changes in RIN for samples stored in −80°C up to a month. We also found that archived microdissected renal cells which were stored in −80°C for six months had very poor RIN of 2.2. Thus, it is highly encouraged to use fresh samples when possible.

We also assessed the RIN of RNA at progressive stages of sample preparation. As listed in Table 2, fresh rat kidney section without embedding and staining generated a RIN of 8.0. It was shown that OCT embedding did not have any impact on RIN. However, RIN dropped to 7.0 after five minutes of the shortened H&E staining, suggesting a certain degree of RNA degradation. However this was still in the acceptable range for qPCR.

**Table 2:** RIN of tissue sections or PTCs at progressive stages of sample preparation

**Experimental conditions**	**RIN**
Fresh tissue section without OCT embedding and H&E stain	8
Fresh tissue section with OCT embedding but without H&E stain	8.1
Fresh tissue scrape with OCT embedding and H&E stain	7
Tissue sections stored in −80°C for a day	7.5
Tissue sections stored in −80°C for a week	6.5
Tissue sections stored in −80°C for 1 month	6
Tissue sections stored in −80°C for 6 months	2.2
PTCs collected within 15 minutes after H&E staining	6.2
PTCs collected within 35 minutes after H&E staining	1

Approximately 4000 renal PTCs which were collected within twenty minutes had a RIN of 6.2. We attempted to collect more renal PTCs within 40 minutes but were unsuccessful as the increased time cased RIN to be degraded to 1.0. Thus, two HS caps of 4000 renal PTCs were collected individually within 20 minutes for a sample and placed on dry ice before pooling together during RNA isolation stage.

#### Modified DNase I treatment

Using the standard DNase I treatment, we observed a Ct value of 27.10 for genomic DNA, suggesting the potential of genomic DNA contamination. Therefore, modifications were made to the existing DNase I treatment as elaborated under Methods section. The Ct value for genomic DNA was then increased to 40 after the changes made in DNase I treatment.

**Figure 3:**
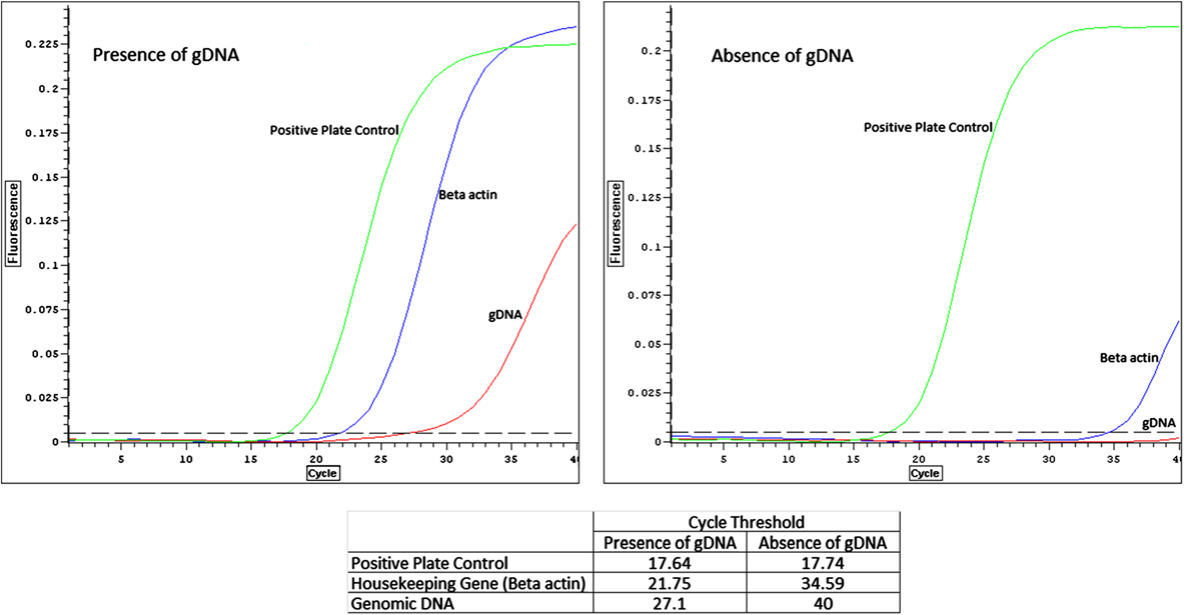
**Effects of DNase I treatment modifications on Ct values.** It was shown that Ct value of gDNA control increased (i.e. gene expression reduced) after modified DNase I treatment was applied. On the other hand, the modified DNase I treatment did not affect the Ct values of positive plate control.

As seen in Figure 3, the Ct value of positive plate control did not change, suggesting that the modified DNase I treatment did not have an effect on the performance of qPCR array. However, there was a change in the Ct value of the housekeeping gene (beta-actin)’s Ct value from 21.75 to 34.59. This shift in Ct value may indicate the true expression value of the gene due to the removal of genomic DNA after application of new DNase I treatment.

#### Application of extracted total RNA on qPCR

The fully optimised protocol was then applied on a total of six rats for the collection of renal PTCs via LCM and subsequent total RNA extraction using commercially available kit. Table 3 below contains the various numbers of renal PTCs, purity ratios, total RNA yields and RIN numbers from each rat. The total RNA extracted from each rat was converted to cDNA, underwent preamplification and qPCR according to manufacturer’s protocol.

**Table 3:** Total renal PTCs, purify ratios, RNA yields and RIN of rat samples

**Rat ID**	**Total renal PTCs collected**	**260/280**	**260/230**	**RNA [ng/μL]**	**RIN**
1	5330	2.03	1.71	2.1	4.7
2	7463	1.83	1.71	2.5	6.2
3	10064	1.81	1.69	3.2	5.7
4	9239	1.74	1.61	3.9	4.9
5	10766	1.92	1.91	2.7	Not available
6	8640	1.97	1.78	2.4	Not available

**Figure 4:**
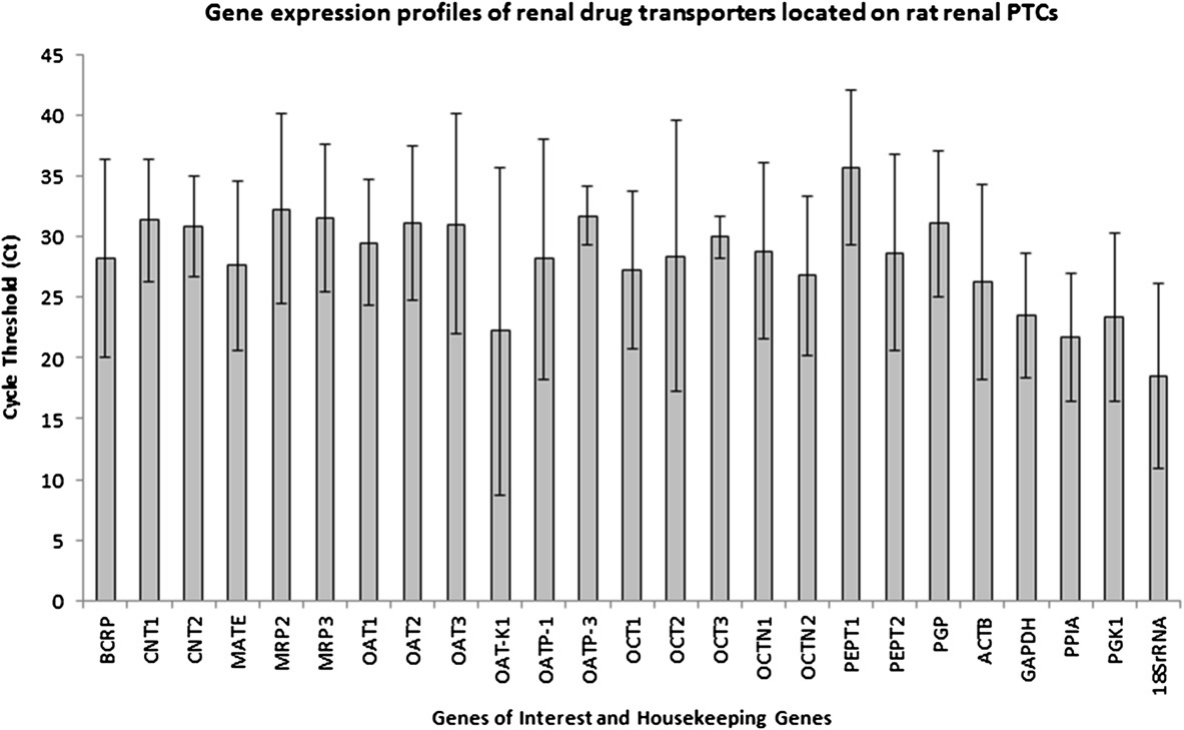
**Gene expression profiles of renal drug transporters located on rat renal PTCs.** All twenty renal drug transporters and five housekeeping genes (ACTB, GAPDH, PPIA, PGK1 and 18SrRNA) had Ct values lower than 35 which confirmed the expression of these selected drug transporters on rat renal PTCs.

A panel of twenty drug transporters which were previously reported to be expressed in rat kidneys were studied. Figure 4 showed the average Ct values of six rats used. Rats 5 and 6 did not have their RIN measured due to unavailability of instrument. All twenty drug renal drug transporters and five housekeeping genes had Ct values below 30, confirming that the expression of such drug transporters on rat renal PTCs can be detected under our experimental conclusions.

### Discussion

One of the greatest challenges when using LCM for qPCR is to collect sufficient RNA of good quality. It was made difficult due to upstream sample preparation procedures which exposed the total RNA to RNases. RNases are found in all cell types and organisms and also commonly detected in typical laboratory settings such as reagents used, environmental exposure and human contact. In order to overcome these issues, we have optimised the progressive steps leading to the collection of total RNA from rat renal PTCs.

A histological stain is required for the efficient identification of rat renal PTCs, and at the same time, minimizing RNA degradation. We attempted to condense the time taken for H&E staining from 26 minutes to 5 minutes. It was then observed that the modified H&E staining not only allowed straightforward identification of rat renal PTCs, and also a minimal impact on RIN.

We also agreed with publications that LCM should be carried out immediately after H&E staining. We later evaluated the RIN of total RNA collected at the progressive stages of sample preparation. We then observed that RIN of rat renal PTCs collected within 15 minutes had a significant better RNA quality than rat renal PTCs collected within 35 minutes. The RIN of an archived rat kidney was extremely poor at 2.2, recommending the usage of fresh kidney tissues to be studied.

As different cell types have different amount of RNA yield, it is important to have a protocol specifically designed for different cell types [5]. We looked into the minimal number of renal PTCs to isolate sufficient total RNA for qPCR. We collected three sets of renal cells, consisting of 3000, 8000 and 18000 cells respectively. Although the RNA 6000 Pico kit was not developed to be a quantitative assay, it was nonetheless used to estimate the RNA concentrations of the collected renal cells due to its low qualitative range (50 to 5000 pg/μL). It was estimated that 8000 and 18000 renal cells generated similar total RNA yields. As no information was provided with regards to the total RNA capacity limit of PicoPure® RNA Isolation Kit (Life Technologies), the similar total RNA yields isolated from 8000 and 18000 renal cells may suggest a possible capacity limit to the RNA isolation kit. Thus, we concluded that at least 8000 rat renal PTCs were required to generate the minimal concentration for qPCR analyses. However, it is important to note that only 4000 rat renal PTCs were able to be collected within the 15 minutes limit. Therefore, two HS caps consisting 4000 rat renal PTCs each were collected for each rat sample. The HS caps containing rat renal PTCs were placed on dry ice to minimise RNase degradation before pooling together during total RNA isolation.

DNase I treatment is often crucial in qPCR analyses. It aids in the elimination of genomic DNA contamination which may contribute to a higher gene expression than its true value. We observed that small modifications made to the existing DNase I treatment as recommended by manufacturer aided in removing more genomic DNA from total RNA isolated. We doubled the amount of DNase I units and the incubation period. It was shown that the Ct values for genomic DNA control increased from 27.10 to 40, indicating a significant reduction in genomic DNA contamination.

The above optimised protocol for extracting total RNA from microdissected rat renal PTCs showed that the amount of total RNA recovered from the cells depends not only on the cell type or size but also the isolation method and/or treatments used.

The above modified techniques were then applied in a total of six Wistar male rats. We were able to collect an average of 8000 rat renal PTCs all six rats. The total RNA concentrations and purity ratios were measured using NanoDrop 2000 and RIN were analysed via RNA 6000 Pico kit. The total RNA extracted from each rat was applied onto qPCR array in the gene expression analysis of twenty drug transporters and five housekeeping genes. The average Ct values of respective genes were below 35 indicating a measurable gene expression level according to manufacturer’s protocol. Therefore, we concluded that the optimised techniques were suitable for a more meaningful qPCR analysis.

These techniques may be applicable for scientists looking into similar cell groups, such as rat renal distal tubular cells or glomerulus. However, researchers may require paying attention on the number of required cells for sufficient RNA concentration and the organs or cells of interest which may possess different levels of RNases, thus requiring more attention to the protocols Microarray study is currently a popular choice for the understanding of gene expression of a huge pool of genes simultaneously. There are several studies conducted for the preparation of RNA from microdissected cells for microarray analyses [18,19]. The criteria for quality and quantity of total RNA from selected cells appeared to be more stringent, i.e. 18S and 28S rRNA ratios and area, a cut off of RIN was set. Although researchers may apply similar techniques such as the identification of renal cells and DNase I treatment, it is important to note on the RNA quality control for microarray analysis. Nonetheless, current study may serve as a guideline for the minimization of false positive qPCR data caused by genomic DNA contamination and RNA degradation by RNases.

### Conclusions

In this study, we described a protocol for the isolation of good quality RNA from microdissected rat renal PTCs for the application of qPCR analyses. In specific, we have optimised a few important procedures: 1) modified shortened H&E staining, 2) minimal number of rat renal PTCs required for qPCR analyses, 3) the quality control of total RNA, and 4) modified DNase I treatment for a more efficient removal of genomic DNA contamination. Using this optimized protocol, we are able to achieve sufficient good quality total RNA from microdissected rat renal PTCs for gene expression profiling via qPCR.

## Competing interests

The authors declare that they have no competing interests.

## Authors’ contributions

JYY and Dr. LMGL optimized the described total RNA isolation method and DNase I treatment, measured the RNA RIN and purity ratios and performed qPCR gene expression profiling. KR, SMR and VSYL performed the tissue preparations, cryosectionings, LCM and input helpful suggestions in the optimizations of protocols. Professor EJDL is the principle investigator of this study shared invaluable experience and guidance in this study. All authors read and approved the final manuscript.
